# Pulmonary Co-Infections Detected Premortem Underestimate Postmortem Findings in a COVID-19 Autopsy Case Series

**DOI:** 10.3390/pathogens12070932

**Published:** 2023-07-12

**Authors:** Andrew P. Platt, Benjamin T. Bradley, Nadia Nasir, Sydney R. Stein, Sabrina C. Ramelli, Marcos J. Ramos-Benitez, James M. Dickey, Madeleine Purcell, Shreya Singireddy, Nicole Hays, Jocelyn Wu, Katherine Raja, Ryan Curto, Stephen J. Salipante, Claire Chisholm, Stephanie Carnes, Desiree A. Marshall, Brad T. Cookson, Kevin M. Vannella, Ronson J. Madathil, Shahabuddin Soherwardi, Michael T. McCurdy, Kapil K. Saharia, Joseph Rabin, NIH COVID-19 Autopsy Consortium, Alison Grazioli, David E. Kleiner, Stephen M. Hewitt, Joshua A. Lieberman, Daniel S. Chertow

**Affiliations:** 1 Emerging Pathogens Section, Critical Care Medicine Department, Clinical Center, National Institutes of Health, Bethesda, MD 20892, USA; andrew.platt@nih.gov (A.P.P.);; 2 Laboratory of Virology, National Institute of Allergy and Infectious Diseases, Bethesda, MD 20892, USA; 3 Department of Pathology, University of Utah, Salt Lake City, UT 84112, USA; 4 Laboratory of Pathology, Center for Cancer Research, National Cancer Institute, National Institutes of Health, Bethesda, MD 20892, USA; 5 Department of Basic Sciences, Division of Microbiology, Ponce Research Institute, School of Medicine, Ponce Health Sciences University, Ponce, PR 00716, USA; 6 University of Maryland School of Medicine, Baltimore, MD 21201, USAdrmccurdy@gmail.com (M.T.M.); 7 Department of Laboratory Medicine and Pathology, University of Washington Medical Center, Seattle, WA 98195, USA; 8 American Esoteric Laboratories, Memphis, TN 38134, USA; 9 Department of Surgery, Division of Cardiac Surgery, University of Maryland School of Medicine, Baltimore, MD 21201, USA; 10 Hospitalist Department, TidalHealth Peninsula Regional, Salisbury, MD 21801, USA; 11 Department of Medicine, University of Maryland St. Joseph Medical Center, Towson, MD 21204, USA; 12 Institute of Human Virology, Division of Infectious Diseases, University of Maryland School of Medicine, Baltimore, MD 21201, USA; 13 R Adams Cowley Shock Trauma Center, Department of Surgery and Program in Trauma, University of Maryland School of Medicine, Baltimore, MD 21201, USA; 14 R Adams Cowley Shock Trauma Center, Department of Medicine and Program in Trauma, University of Maryland School of Medicine, Baltimore, MD 21201, USA

**Keywords:** COVID-19, pneumonia, co-infection, invasive fungal infections, autopsy, nosocomial infections

## Abstract

Bacterial and fungal co-infections are reported complications of coronavirus disease 2019 (COVID-19) in critically ill patients but may go unrecognized premortem due to diagnostic limitations. We compared the premortem with the postmortem detection of pulmonary co-infections in 55 fatal COVID-19 cases from March 2020 to March 2021. The concordance in the premortem versus the postmortem diagnoses and the pathogen identification were evaluated. Premortem pulmonary co-infections were extracted from medical charts while applying standard diagnostic definitions. Postmortem co-infection was defined by compatible lung histopathology with or without the detection of an organism in tissue by bacterial or fungal staining, or polymerase chain reaction (PCR) with broad-range bacterial and fungal primers. Pulmonary co-infection was detected premortem in significantly fewer cases (15/55, 27%) than were detected postmortem (36/55, 65%; *p* < 0.0001). Among cases in which co-infection was detected postmortem by histopathology, an organism was identified in 27/36 (75%) of cases. *Pseudomonas*, *Enterobacterales*, and *Staphylococcus aureus* were the most frequently identified bacteria both premortem and postmortem. Invasive pulmonary fungal infection was detected in five cases postmortem, but in no cases premortem. According to the univariate analyses, the patients with undiagnosed pulmonary co-infection had significantly shorter hospital (*p* = 0.0012) and intensive care unit (*p* = 0.0006) stays and significantly fewer extra-pulmonary infections (*p* = 0.0021). Bacterial and fungal pulmonary co-infection are under-recognized complications in critically ill patients with COVID-19.

## 1. Introduction

Bacterial co-infection commonly complicates severe viral pneumonias. A retrospective analysis of preserved lung specimens from fatal 1918 influenza cases revealed evidence of co-infection in 95% of the cases [1]. In the post-antibiotic era, the frequency of bacterial co-infection detected postmortem has declined, although it remains a significant burden [2]. For example, in a series of fatal 2009 H1N1 influenza cases, postmortem bacterial co-infection was detected in 26% of cases [3]. The frequency of co-infection in coronavirus disease 2019 (COVID-19) is less well characterized than influenza, but it is likely to vary by disease severity and other relevant demographic and clinical factors [4,5]. Among patients initially presenting to the hospital for COVID-19, bacterial co-infection was reported in approximately 2% of cases [6], compared with 37–58% of cases among more severely ill hospitalized or mechanically ventilated patients [7,8]. Here, we define pulmonary co-infection as bacterial or fungal infections occurring concurrently with the initial SARS-CoV-2 infection, as opposed to superinfections occurring after COVID-19 has resolved [9].

Pulmonary co-infection is a significant burden for critically ill patients with COVID-19 and has been associated with worse outcomes [10,11]. In critically ill patients, hospital-acquired infections (HAIs) have been shown to predominate, including high rates of nosocomial and multidrug-resistant (MDR) bacteria [12]. The diagnostic differentiation of co-infection from the progression of COVID-19 pneumonia and acute respiratory distress syndrome (ARDS) or other pulmonary process remains challenging, although imaging may help in the diagnosis [13]. In a meta-analysis of fatal COVID-19 cases in which an autopsy was performed, bacterial co-infection was reported in 200 of 621 (32%) cases, predominantly with nosocomial pathogens including *Escherichia coli*, *Acinetobacter baumannii*, *Pseudomonas aeruginosa*, and *Klebsiella pneumoniae* [14,15]. However, co-infection is often only incidentally noted, and it was a primary endpoint in only 3/75 (4%) of studies [14].

The phenomenon of COVID-19-associated pulmonary aspergillosis (CAPA) has been described in multiple case series [16,17,18]. The assessment of the burden of CAPA has been complicated by a lack of standard case definitions and the infrequent pursuit of tissue-based diagnoses for critically ill patients [19]. The estimates of CAPA in registry studies range from 1–39.1% [20,21], and further work is needed to establish the true burden of disease in COVID-19.

We therefore sought to determine the burden of pulmonary co-infection in fatal cases of COVID-19, whether premortem diagnoses of pulmonary co-infection accurately predicted postmortem findings, the prevailing pathogens, and whether there was concordance between the species identified pre- and postmortem. Among 55 fatal COVID-19 cases from two medical centers in the United States from March 2020 to March 2021, we observed that bacterial and fungal co-infection detected premortem underestimated the burden of disease detected postmortem.

## 2. Materials and Methods

### 2.1. Autopsies

Autopsies were performed and tissues collected as previously described [22,23]. Those for cases P1–P44 were performed at the National Cancer Institute’s Laboratory of Pathology at the National Institutes of Health (NIH) Clinical Center (Bethesda, MD, USA), coordinated by the NIH COVID-19 Autopsy Consortium and with consent of the legal next of kin. Autopsies for cases AU-10 through AU-31 were performed at the King County Medical Examiner’s Office (Seattle, WA, USA) and the University of Washington (UW) (Seattle, WA, USA). The case series was a convenience sample of all cases referred to these institutions for autopsy during the sample period. A total of 55 cases with proven SARS-CoV-2 testing were included with no exclusion criteria. Due to biosafety concerns related to potential severe acute respiratory syndrome coronavirus SARS-CoV-2 laboratory exposures, bacterial and fungal cultures were not performed at the time of autopsy.

### 2.2. Histopathology

Standard histopathologic stains, including hematoxylin and eosin (H&E), Brown and Hopps (B&H), and Gomori methenamine silver (GMS), were performed on lung tissues from all lobes of all patients. Acute bronchopneumonia on histology (i.e., the presence of neutrophil infiltrates in alveolar spaces and bronchioles) was used to diagnose pulmonary co-infection, as this finding is not observed in the diffuse alveolar damage (DAD) pattern seen in COVID-19 pneumonia alone [22,23]. Cases demonstrating acute bronchopneumonia were classified as positive for a microorganism following a positive result by special stains or PCR and sequencing.

### 2.3. Chart Review

Medical records from all cases were independently reviewed by two members of the research team. Patient demographics and treatments were recorded using a standardized form (Supplementary Figure S1). Co-morbidities previously associated with poor outcomes in COVID-19 were recorded [24]. Premortem pulmonary co-infection was determined on retrospective chart review using criteria previously validated in critically ill patients with respiratory insufficiency or failure [25] and consistent with society guidelines for the diagnosis of hospital-acquired and ventilator-associated pneumonias (HAP/VAPs) [26]. Briefly, this included radiographically confirmed lung infiltrates plus two or more of the following criteria: new fever, new leukocytosis, and new purulent pulmonary secretions. In cases in which the above could not be adequately determined on chart review, increasing oxygen requirements in a previously stable patient was used as an additional criterion. Due to conflicting diagnoses between primary and consulting teams and frequent use and discontinuation of empiric antibiotics, clinical documentation and antibiotic use were not used as diagnostic criteria. For all cases that met criteria for premortem pulmonary co-infection, respiratory culture data from the time of diagnosis were reviewed. If cultures grew one or more pneumopathogens, these were considered the causative agents. Diagnosis of an extra-pulmonary co-infection was made by (a) positive culture of a pathogen from a normally sterile site or (b) positive culture from a non-sterile site plus signs and symptoms consistent with infection. Nosocomial pathogens and antibiotic regimens appropriate for HAP/VAPs were defined according to society guidelines [26]. Briefly, this required an agent covering methicillin-resistant *Staphylococcus aureus* and Gram-negative coverage with an anti-pseudomonal agent. Patients were defined as having “diagnosed pulmonary co-infection” if they had premortem and postmortem diagnoses of pulmonary co-infection, “undiagnosed pulmonary co-infection” if they had no premortem diagnosis of pulmonary co-infection but did have a postmortem diagnosis of co-infection, and “no pulmonary co-infection” if they had no postmortem pulmonary co-infection, regardless of premortem diagnoses.

### 2.4. Statistics

All statistics were calculated in Prism 8 (GraphPad, San Diego, CA, USA). Continuous variables were compared using a two-tailed *t*-test for normally distributed variables and a two-tailed Mann–Whitney test for non-normal variables between two groups and one-way ANOVA or Kruskal–Wallis test for normal and non-normal variables between three or more groups. Dichotomous variables were compared using the Fisher exact test or chi-squared test. A *p*-value of <0.05 was used to define significance.

### 2.5. Pathogen Identification by PCR and Sequence-Based Taxonomic Classification

Both formalin-fixed paraffin-embedded (FFPE) and frozen, unfixed tissues were investigated for the presence of bacterial and/or fungal pathogens by clinically validated, laboratory-developed tests in the CLIA-certified, high-complexity clinical molecular microbiology laboratory at UW. Briefly, DNA was extracted from FFPE tissue with the FFPE Advanced Kit (Qiagen, Hilden, Germany) following the manufacturer’s instructions. The DNA was extracted from unfixed tissue as previously described [27]. The PCR amplifications were performed as previously described: broad-range bacterial PCR targeted the V1–V2 hypervariable region of the 16S rRNA gene [28], and broad-range fungal PCR targeted the 28S D1/D2, ITS1, and ITS2 loci [29,30]. Amplified products were sequenced, and taxonomic classification was assigned by BLAST analysis [31] using both public databases (NCBI) and an in-house proprietary database, with priority given to type strain and refseq records, as previously described [27,32].

When sequencing of 16S products suggested multiple bacterial templates, bacterial populations were deconvoluted using amplicon next-generation sequencing (NGS) on an Illumina Miseq with 250 bp paired-end reads, as previously described [33].

## 3. Results

### 3.1. Case-Series Demographics

Fifty-five postmortem COVID-19 cases were enrolled between 27 March 2020 and 2 March 2021 from NIH and UW. In total, 18/55 (33%) patients were female. The median patient age was 63 (IQR 48.5–71). In total, 22/55 (45%) patients identified as White, 19/55 (35%) identified as Black, 11/55 (20%) identified as Hispanic, and 1/55 (2%) identified as Asian. The median body-mass index (BMI) was 31.2 (IQR 25.9–36.8), and 49/55 (89.1%) patients had at least one major comorbidity (Table 1). No significant differences in demographic or clinical variables were detected between the UW and NIH cases, except that the UW cases had significantly longer postmortem intervals (median 72 vs. 22 h, *p* < 0.0001, Supplementary Table S1).

### 3.2. Clinical Interventions

In total, 51/55 (92.7%) patients were admitted to an intensive care unit (ICU) for a median of 12 days (IQR 5.5–23.5), 46/55 (83.6%) received invasive mechanical ventilation for a median of 11 days (IQR 2.75–21.25), 44/55 (80.0%) required vasopressor support, 22/55 (40.0%) required renal replacement therapy, and 13/55 (23.6%) were placed on extra-corporeal membrane oxygenation (ECMO) (Table 2). Furthermore, 50/55 (90.9%) patients received at least one dose of an antibiotic during their hospital admission, 46/55 (83.6%) patients received a corticosteroid, 24/55 (43.6%) received the antiviral drug remdesivir, 4/55 (7.2%) received the anti-IL-6 monoclonal antibody tocilizumab, and 7/55 (12.7%) received convalescent plasma.

### 3.3. Premortem Co-Infections

In total, 15/55 (27.3%) patients had a pulmonary co-infection diagnosed premortem, of which three had only a pulmonary co-infection and 12 had both pulmonary and extra-pulmonary co-infections. Furthermore, 23/55 cases (41.8%) met the criteria for any co-infection diagnosed premortem at any site, including the lungs, blood stream, urinary tract, and skin and soft tissue, as well as *Clostridium difficile* colitis (Table 3). The most common extra-pulmonary co-infection was bacteremia (16/55, 29.1%). In 7/12 cases with both pulmonary and extra-pulmonary (six bacteremia, one hepatic abscess) co-infections identified premortem, the same organism was recovered from the cultures from both sites.

### 3.4. Postmortem Co-Infection Findings

**Figure 1:**
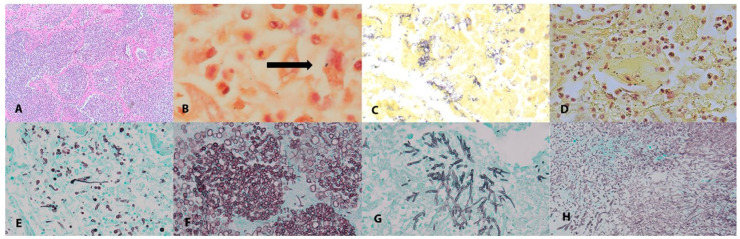
Histological examination of lung tissue. (**A**) Hematoxylin and eosin (**E**,**H**) staining demonstrating bronchopneumonia with neutrophils within the alveolar spaces in P30, 40× magnification. (**B**–**D**) Brown–Hopps stain of lung sections. (**B**) Rare diplococci (indicated by arrow) in P8, 100× magnification. (**C**) Gram-negative rods in P11, 60× magnification. (**D**) Gram-positive cocci in pairs in P30, 60× magnification. (**E**–**H**) GMS stain highlighting fungal organisms of lung sections. (**E**,**F**) Multiple foci of yeasts and pseudo hyphae in P1 and P5 at 40× magnification. (**G**) Cluster of fungal hyphae consistent with underlying *Aspergillus* infection in P15, 40× magnification. (**H**) Fungal ball composed of yeasts and hyphae in P44, 40× magnification.

At autopsy, 36/55 cases (65.5%) had evidence of pulmonary co-infection (Table 1, Figure 1). A positive bacterial stain was observed in 25/36 (69.4%) of these cases and a positive GMS stain consistent with invasive fungal infection was observed in five cases (Figure 1).

### 3.5. Comparison of Premortem and Postmortem Pulmonary Infection Findings

There were significantly more cases with a postmortem diagnosis of pulmonary co-infection (36/55, 65.5%) than were diagnosed premortem (15/55, 27.2%, *p* = 0.0001). In total, 32/55 cases (58.2%) had a postmortem diagnosis of pulmonary co-infection and evidence of a pathogen, which was also significantly higher than the proportion of cases diagnosed with pulmonary co-infection premortem (15/55, 27.2%, *p* = 0.0019).

### 3.6. Factors Associated with Premortem Identification of Pulmonary Infection

Of the 36 cases with a postmortem diagnosis of pulmonary co-infection, 11/36 (30.6%) had a diagnosed pulmonary co-infection, while 25/36 (69.4%) had an undiagnosed pulmonary co-infection (Table 3). The patients with undiagnosed pulmonary co-infections were significantly less likely to be Hispanic (2/25 vs. 6/11, *p* = 0.0048), had lower BMI (median 31 vs. 39, *p* = 0.0368), and tended to be older (median 63 vs. 48, *p* = 0.0995) (Table 1). The patients with undiagnosed pulmonary co-infections also had significantly shorter times from symptom onset to death (median 16 vs. 48 days, *p* = 0.0009) and shorter hospital (median 8 vs. 45 days, *p* = 0.0012) and ICU stays (median 7 vs. 44 days, *p* = 0.0006), and spent less time on ventilators (median 4 vs. 42 days, *p* = 0.0003). The rates of ICU admission and intubation were similar between the patients with diagnosed and undiagnosed pulmonary co-infections, while the patients with diagnosed pulmonary co-infections were significantly more likely to be placed on an ECMO circuit (6/11 vs. 1/25, *p* = 0.0022) (Table 2). While the rates of antibiotic use were high in both groups (11/11 vs. 23/25) there was a strong trend towards lower usages of combinations of antibiotics appropriate for the coverage of hospital-acquired or ventilator-associated pneumonia in patients with undiagnosed pulmonary co-infections (14/25 (56%) vs. 10/11 (91%), *p* = 0.0828) [26]. Patients with diagnosed pulmonary co-infections were more likely to be diagnosed with premortem extrapulmonary infections (9/11 vs. 2/25, *p* < 0.0001) (Table 3).

### 3.7. Identification of Pre- and Postmortem Pulmonary Bacterial Pathogens

To identify the bacterial pathogens from postmortem specimens, we attempted 16S PCR and sequencing (Supplementary Figure S2). We first sequenced from one FFPE block of lung tissue, from a lobe in which acute bronchopneumonia was seen, for each of the 36 cases with a postmortem diagnosis of pulmonary co-infection. This approach identified a pathogen in only six cases. To increase the recovery of intact nucleic acids for sequencing, we repeated the analysis with frozen tissue taken adjacent to histopathologically confirmed bronchopneumonia. Of the 30 cases with preliminarily negative results, tissues were available for 22. The sequencing of the frozen samples was positive in 10 cases, for a total of 16 positive sequencing results.

**Figure 2:**
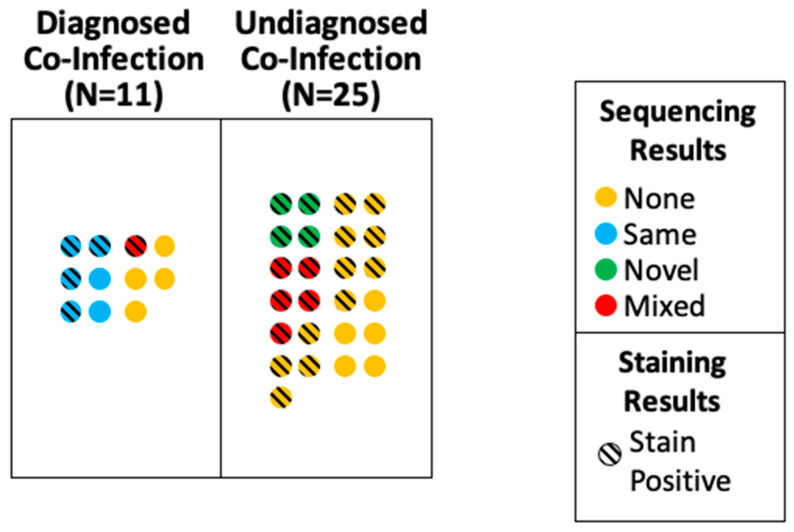
Pathogen identification in cases with diagnosed and undiagnosed pneumonia, and correlation between premortem culture results and postmortem sequencing results. Each colored circle represents one case. Yellow: no PCR product available for sequencing. Blue: postmortem sequencing identified the same species of pathogen as premortem respiratory culture. Green: postmortem sequencing identified an organism not seen on premortem culture. Red: postmortem sequencing identified mixed flora. Diagonal black lines indicate positive B&H stain on histology.

From the premortem positive cultures, the most frequently isolated pathogens were *p. aeruginosa* (*n* = 4) and *K. pneumoniae* (*n* = 4)*,* followed by methicillin-susceptible *Staphylococcus aureus* (MSSA) (*n* = 3) and methicillin-resistant *Staphylococcus aureus* (MRSA) (*n* = 3). In the postmortem sequencing, *E. coli* (*n* = 3) and *P. aeruginosa* (*n* = 2) were the most frequently identified pathogens (Table 4). In cases in which a pathogen was identified both pre- and postmortem, the same pathogen was identified in six of seven cases (Figure 2). While most of the organisms identified by the sequencing were plausible pneumopathogens, for patient P9, *P. aeruginosa* and *Enterococcus faecium* were found by 16S PCR and sequencing. This patient had premortem diagnoses of *P. aeruginosa* VAP and *E. faecium* bacteremia. Therefore, we performed 16S PCR and sequencing using the liver tissue, through which we identified only *E. faecium*, which is consistent with bacteremia. A 16S PCR was performed on five cases without pulmonary co-infection, and all the cases were negative. The NGS sequencing reports are available in Supplementary Table S2.

### 3.8. Pulmonary Fungal Co-Infections

Invasive fungal pulmonary infections were identified on histology in 5/55 (9.1%) cases (Figure 1). Cases in which yeast were seen within the airways but without evidence of invasive disease were excluded. Case P15 demonstrated septate hyphae on GMS staining consistent with a septate hyaline mold without a premortem diagnosis of fungal infection or anti-fungal coverage. This patient had a history of sarcoidosis but was not on immunosuppression prior to admission. Cases P5 and P39 had invasive yeast and pseudohyphae; both cases had premortem diagnoses of fungemia (*Candida albicans* and *Candida lusitaniae,* respectively). Case P1 had invasive yeast and pseudohyphae without a premortem diagnosis of fungal infection or anti-fungal coverage. Case P44 had invasive yeast and pseudohyphae in one lobe and a fungal ball with septate branching forms in a second lobe, and had been diagnosed premortem with a *C. albicans* empyema. Cases P5, P15, P39, and P44 all received extended methylprednisolone tapers in addition to initial courses of steroids. Antifungal coverage was given for the three cases in which a fungal infection at a different site was identified premortem, plus an additional five cases that did not have evidence of fungal disease. All the foci of invasive disease were limited to small, adjacent alveolar spaces rather than being widely disseminated. All the cases of invasive fungal infection were identified only in FFPE blocks that had been fixed for >72 h. The PCRs of the 28s and ITS loci performed on the DNA extracted from the highly fixed blocks and from the blocks from remote locations in the same lobe fixed for only 24 h failed to yield a product that could be sequenced. In no case was frozen tissue immediately adjacent to a positive GMS stain available to attempt sequencing from unfixed tissues.

All five cases of pulmonary fungal co-infection had evidence of acute bronchopneumonia on histology. Additionally, four of these five cases had a B&H stain positive for bacteria, while P5 had a premortem diagnosis of bacterial co-infection with cultures positive for *K. pneumonia* and *S. marcescens* (Supplementary Table S2). To identify the risk factors for invasive fungal pulmonary co-infection, we compared these five cases with all the cases with bacteria-only pulmonary co-infection across all the demographic and clinical factors reported (Supplementary Table S3). The patients with fungal co-infection were significantly younger (median age 41 vs. 63, *p* = 0.048) and had fewer comorbidities (median 1 vs. 2, *p* = 0.0273) than those with only bacterial infections. The fungal cases had significantly longer times from symptom onset to death (median 31 vs. 18 days, *p* = 0.0453), as well as longer hospital and ICU stays (median 27 vs. 10 days, *p* = 0.0016 and 22 vs. 7 days, *p* = 0.0207, respectively).

## 4. Discussion

In a case series of 55 COVID-19 autopsy patients, we found that a higher proportion of patients had a pulmonary co-infection than met a set of standardized clinical criteria for diagnosis premortem. The proportion of cases with pulmonary co-infection at autopsy was significantly higher (*p* < 0.0001) than the 200/621 (32%) reported in a prior meta-analysis [14]. The identified pathogens were predominantly nosocomial and showed good agreement between premortem cultures and postmortem sequencing. The clinical factors associated with undiagnosed pulmonary co-infection premortem included shorter length of stay, decreased use of ICU resources, including ECMO, and the absence of extra-pulmonary co-infections. These results suggest that pulmonary co-infections may be initially under-diagnosed in a subset of critically ill patients in the initial period after admission. This may be due to a shorter interval for diagnostic consideration, a lower index of suspicion in newly admitted patients, and the less frequent monitoring of patients receiving lower-intensity care.

In comparison to other studies, our use of acute bronchopneumonia as the primary histopathologic endpoint and our thorough examination of all the lung lobes from all the patients may have increased the sensitivity of the postmortem detection of co-infection; in over 96% of other autopsy case series, bronchopneumonia was only incidentally reported [14]. The cases we presented were from the first year of the pandemic and may represent the evolution of practice patterns that were not in place during the first months of the pandemic and that were less likely to be affected by short-term effects, such as surge conditions. The similarity between the rates of pulmonary co-infection observed in the NIH and UW cases suggests that the high rate of co-infection was not driven by local medical practices.

Despite the high rate of intubation among our cases, VAP alone does not explain the proportion of patients with pulmonary co-infection: based on previously published rates of VAP in the United States, we would expect 1–3 cases per 1000 ventilator days, or only 1.8–3.6 cases of VAP within our case series, rather than the 36 observed [34,35]. The case rate of 65.5% was also higher than those reported in autopsy series of the contemporary H1N1 influenza pandemic [36]. It is not clear whether the higher proportion of pulmonary co-infection in our case series was due to intrinsic SARS-CoV-2-mediated factors, such as the impairment of the myeloid compartment [37], or to clinical-practice patterns during the epidemic, such as high rates of steroid usage and immunomodulatory therapy.

The diagnosis of lower-respiratory-tract infections is known to be challenging in scenarios with concomitant lung pathology, and no proposed sets of clinical and/or microbiological markers have shown excellent sensitivity and specificity when compared to histology [25]. Using the presence of bronchopneumonia on histology as the gold standard, the clinical criteria used to diagnose pulmonary co-infections had a sensitivity of 30.6% and a specificity of 78.9% in this study. Probably due to the progression of viral pneumonia and ARDS, new infiltrates and consolidations were present in almost all the patients on chest X-ray, regardless of the findings on autopsy, suggesting that this criterion had poor specificity. Cross-sectional imaging may offer a greater ability to resolve disparate disease processes [13]. The poor performance of these clinical criteria is in line with the findings reported in other critically ill patients [25], and the low sensitivity in this series of fatal cases suggests that there should be a high index of suspicion for pulmonary co-infection among critically ill COVID-19 patients.

In prior studies on COVID-19, the identification of co-infecting organisms primarily relied upon the correlation of culture results with histology or appearance on special stains. Tissue-based molecular-pathogen detection has demonstrated excellent clinical sensitivity and the ability to distinguish morphologically identical organisms [38]. These results support the use of molecular techniques in postmortem studies as a valuable adjunct for premortem infectious-disease studies, although the yield of 16/36 (44%) of cases with a PCR product available for sequencing does represent a limitation. The increased yield of sequencing data using frozen tissue highlights the importance of collecting frozen tissue at autopsy. In two cases (P20 and AU-21), no organisms were seen on special stains, but an organism was identified by sequencing. In both cases, the organism (*P. aeruginosa* and *S. aureus*, respectively) was correlated with a positive premortem respiratory culture, supporting the validity of our approach. In cases P8 (Figure 1) and P14, only rare organisms that were not sufficient to identify a pathogen were seen on the special stains; these were identified through sequencing as *A. baumannii* and *Legionella* sp., respectively. The risk of the misdiagnosis of postmortem bacterial overgrowth was minimized by short postmortem intervals and the need for the simultaneous presence of acute bronchopneumonia.

Most of the bacterial pathogens identified were Gram-negative rods, including lactose non-fermenters and members of the order *Enterobacterales*, which is consistent with nosocomial infections. One organism (P14, *Legionella* sp.) was more likely to be a community-acquired pathogen. The good agreement between the premortem culture and the postmortem sequencing supports the attribution of pulmonary infection to these pathogens and the utility of sequencing in the identification of pathogens in situations in which postmortem culture is not possible. The predominance of Gram-negative rods was consistent with studies on living patients [15] and autopsy series [14]. Based on this finding, empiric antibiotic coverage in critically ill patients with COVID-19 should be tailored toward hospital-acquired rather than community-acquired pathogens. The strong trend towards the greater use of antibiotics appropriate for hospital-acquired infection in patients with diagnosed pulmonary co-infection probably failed to reach significance due to the limited sample size in our study. This is also consistent with prior work from the same geographic region and time period, which found very low rates of bacterial co-infection at the time of hospital admission for COVID-19 [6]. This is in contrast with pre- and postmortem studies of influenza co-infection [1] and suggests differences between the underlying pathogeneses. The implication for antibiotic selection is that for critically ill patients with COVID-19 that show evidence of pulmonary co-infection, antibiotics should be tailored to nosocomial pathogens. As this study only included critically ill, fatal cases of COVID-19, these findings should not be generalized to all cases of COVID-19 or all hospitalized patients, for whom antibiotic prescribing patterns may be unnecessarily high [39,40].

Five cases of invasive fungal pulmonary co-infection were found on autopsy, none of which had been identified premortem, although fungal infection at other sites had been diagnosed in three of the five cases. On average, these patients had longer hospital and ICU stays; this was probably related to their younger age and fewer comorbidities compared with patients who had pulmonary bacterial co-infections without fungal co-infections. This may have been an independent risk factor and may have contributed to greater cumulative antibiotic and/or steroid administration, and it should serve as a factor in the identification of patients at risk of pulmonary fungal co-infection in the future. As recommended treatment durations for invasive fungal infections are generally longer than for bloodstream infections, longer treatment durations for fungal infections in COVID-19 may need to be considered. Two cases (P15 and P44) had septate hyphae on histology that was not identified with culture or sequencing. These findings are consistent with but do not confirm *Aspergillus* spp. and CAPA.

We acknowledge several limitations in our study. There were inherent biases in our postmortem case series, which may have over-represented severe illness and pathology, including selection bias and the lack of a control group. As the recruitment ended before the widespread vaccination for SARS-CoV-2 was available, the cases in this case series were all unvaccinated. A recent analysis of poor outcomes for vaccinated individuals found a median of four major co-morbidities in fatal cases of COVID-19, compared to the median of two in this study [24]. Although we did not observe a higher rate of bronchopneumonia in the patients with more comorbidities, this does suggest that the patient populations may not be directly comparable. The timings of the cases in this case series mean that we cannot draw conclusions regarding currently circulating variants. Next-generation sequencing may be overly sensitive and detect colonizing bacteria, although we noted good agreement between the bacterial stains, sequencing, premortem culture data (when available, and acknowledging the lack of postmortem cultures) and histopathology. Due to the nature of autopsies, we were unable to prove whether the death of a patient with bronchopneumonia was due to COVID-19 or to bacterial/fungal co-infection. Given the complex patterns of antibiotic administration in critically ill patients, it was not possible to categorize antibiotic use more deeply as empiric, targeted towards presumed diagnosis, or definitive. Due to the relatively small sample size of 55 patients, it is not clear whether some of the statistical associations, such as Hispanic ethnicity and diagnosed pulmonary co-infection, were due to sampling or to true pathogenesis.

Despite these limitations, we were able to draw meaningful conclusions from an investigation of bacterial and fungal pulmonary co-infections in a large, multi-center autopsy case series of COVID-19 patients. We found that in our case series, pulmonary co-infection was more common than previously believed, that the rates of pulmonary co-infection were significantly higher than those recognized premortem, that bacterial co-infections were predominantly caused by nosocomial pathogens, and that invasive fungal infections are under-recognized in this population. The patients with undiagnosed pulmonary co-infections had shorter hospital and ICU stays, fewer extra-pulmonary infections, and tended to receive potentially inappropriate antibiotic therapy. These findings suggest that in critically ill patients with COVID-19, there should be high index of suspicion for pulmonary bacterial co-infection, supporting empiric antibiotic use to covering nosocomial pathogens in this population. However, antibiotic stewardship must remain a priority for these patients, and the narrowing or discontinuation of therapy should be guided by culture results and clinical status.

## Figures and Tables

**Table 1:** Demographics of autopsy cases separated by co-infection diagnosis. The *p* values are univariate analyses of the difference between patients with pulmonary co-infection diagnosed pre- and postmortem (diagnosed pulmonary co-infection), patients with pulmonary co-infection diagnosed only postmortem (undiagnosed pulmonary co-infection), and patients with no pulmonary co-infection postmortem regardless of premortem diagnosis (no pulmonary co-infection).

	All Cases (*n* = 55)	Diagnosed Pulmonary Co-Infection (*n* = 11)	Undiagnosed Pulmonary Co-Infection (*n* = 25)	No Pulmonary Co-Infection (*n* = 19)	*p*-Value
Median age in years (IQR)	63 (48.5,71)	48 (41.5,64.5)	63 (60,71)	68 (48.5,73)	0.0995
Female (%)	18/55 (32.7%)	3/11 (27.3%)	9/25 (36.0%)	6/19 (31.6%)	0.2187
Race (%)					0.1384
White (%)	25/55 (45.5%)	4/11 (36.4%)	13/25 (52.0%)	8/19 (42.1%)	
Black or African American (%)	19/55 (34.5%)	2/11 (18.2%)	10/25 (40.0%)	7/19 (36.8%)	
Hispanic Ethnicity (%)	11/55 (20.0%)	6/11 (54.5%)	2/25 (8.0%)	3/19 (15.8%)	0.0048
Median BMI (IQR)	31.15 (25.9,36.8)	39 (34.3,48.7)	31 (24.3,35.4)	28 (25.2,33.45)	0.0368
Major Comorbidities (IQR)	2 (1,3)	1 (1,3)	2 (2,4)	2 (1.5,3)	0.3632
Immunosuppression/Cancer (%)	9/55 (16.4%)	1/11 (9.1%)	4/25 (11.1%)	4/19 (21.1%)	0.7328
Pulmonary (%)	20/55 (36.4%)	4/11 (36.4%)	8/25 (22.2%)	8/19 (42.1%)	0.4764
Cardiac (%)	33/55 (60.0%)	3/11 (27.3%)	19/25 (52.8%)	11/19 (57.9%)	0.0222
Liver (%)	3/55 (5.5%)	0/11 (0.0%)	2/25 (5.6%)	1/19 (5.3%)	0.9501
Neuro (%)	5/55 (9.1%)	1/11 (9.1%)	2/25 (5.6%)	2/19 (10.5%)	0.0834
Diabetes Mellitus (%)	23/55 (41.8%)	3/11 (27.3%)	13/25 (36.1%)	7/19 (36.8%)	0.3304
CKD (%)	9/55 (16.4%)	0/11 (0.0%)	5/25 (13.9%)	4/19 (21.1%)	0.2594
Obesity (%)	30/55 (54.5%)	9/11 (81.8%)	13/25 (36.1%)	8/19 (42.1%)	0.1027

BMI: body-mass index; CKD: chronic kidney disease.

**Table 2:** Treatment characteristics of autopsy cases separated by co-infection diagnosis. The *p* values are univariate analyses of the difference between patients with diagnosed pulmonary co-infection, undiagnosed pulmonary co-infection, and no pulmonary co-infection.

	All Cases (*n* = 55)	Diagnosed Pulmonary Co-Infection (*n* = 11)	Undiagnosed Pulmonary Co-Infection (*n* = 25)	No Pulmonary Co-Infection (*n* = 19)	*p*-Value
Days from onset to death (IQR)	18 (12,32)	48 (33,65.5)	16 (9,19)	23 (10,31)	0.0009
Hospital duration, days (IQR)	12 (6,25.5)	45 (23,59)	8 (5,16)	12 (4.5,26.5)	0.0012
ICU duration, days (IQR)	12 (5.5,23.5)	44 (21,55.5)	7 (2,12)	11 (1,21.5)	0.0006
Intubation duration, days (IQR)	11 (2.8,21.3)	42 (20.5,55.5)	4 (0.0,10.0)	11 (0.0,16.0)	0.0003
Post-Mortem Interval, hours (IQR)	24 (18.9,39.5)	24.6 (18.6,43.5)	24.0 (19.4,35.1)	23.1 (16.0,46.5)	0.8793
ICU Admission (%)	51/55 (92.7%)	11/11 (100.0%)	23/25 (92.0%)	17/19 (89.5%)	0.5542
Intubated (%)	47/55 (85.5%)	11/11 (100.0%)	21/25 (84.0%)	15/19 (78.9%)	0.2777
Pressor Use (%)	44/55 (80.0%)	11/11 (100.0%)	17/25 (68.0%)	16/19 (84.2%)	0.0712
RRT (%)	22/55 (40.0%)	6/11 (54.5%)	9/25 (36.0%)	7/19 (36.8%)	0.5446
ECMO (%)	13/55 (23.6%)	6/11 (54.5%)	1/25 (4.0%)	7/19 (36.8%)	0.0022
Abnormal Chest Imaging (%)	52/55 (94.5%)	10/11 (90.9%)	25/25 (100%)	17/19 (89.7%)	0.2629
Antibiotic Use (%)	50/55 (90.9%)	11/11 (100.0%)	23/25 (92.0%)	16/19 (84.2%)	0.0288
HAP Coverage (%)	34/55 (61.8%)	10/11 (90.9%)	14/25 (56.0%)	10/19 (52.6%)	0.0828
COVID-Specific Therapies					
Steroid Use (%)	46/55 (83.6%)	11/11 (100.0%)	21/25 (84.0%)	14/19 (73.7%)	0.1712
Remdesivir (%)	24/55 (43.6%)	5/11 (45.5%)	13/25 (52.0%)	5/19 (26.3%)	0.2229
Tocilizumab (%)	4/55 (7.3%)	4/11 (36.4%)	0/25 (0.0%)	1/19 (5.3%)	0.0017
Convalescent Plasma (%)	7/55 (12.7%)	5/11 (45.5%)	3/25 (12.0%)	3/19 (15.8%)	0.0589

ICU: intensive care unit. RRT: renal replacement therapy. ECMO: extra-corporeal membrane oxygenation. HAP: hospital-acquired pneumonia. Abnormal chest imaging: any new infiltrative or consolidative process.

**Table 3:** Premortem infections and type or location of infection. Extra-pulmonary infections were further subdivided by site of infection. The *p* values are univariate analyses of the difference between patients with diagnosed pulmonary co-infection, undiagnosed pulmonary co-infection, and no pulmonary co-infection.

	All Cases (*n* = 55)	Diagnosed Pulmonary Co-Infection (*n* = 11)	Undiagnosed Pulmonary Co-Infection (*n* = 25)	No Pulmonary Co-Infection (*n* = 19)	*p*-Value
Any Pre-mortem Infection	23/55 (41.8%)	11/11 (100.0%)	5/25 (20.0%)	7/19 (36.8%)	<0.0001
Pulmonary	15/55 (27.3%)	11/11 (100.0%)	0/25 (0.0%)	4/19 (21.1%)	<0.0001
Extra-Pulmonary	20/55 (36.4%)	9/11 (81.8%)	6/25 (24.0%)	5/19 (26.3%)	0.0021
Bacteremia	16/55 (29.1%)	9/11 (81.8%)	2/25 (8.0%)	5/19 (26.3%)	<0.0001
UTI	4/55 (7.3%)	0/11 (0.0%)	2/25 (8.0%)	2/19 (10.5%)	0.5542
Fungemia	4/55 (7.3%)	2/11 (18.2%)	2/25 (8.0%)	0/19 (0.0%)	0.1781
Skin/Soft Tissue Infection	1/55 (1.8%)	0/11 (0.0%)	1/25 (4.0%)	0/19 (0.0%)	0.5427
*C. difficile* colitis	1/55 (1.8%)	1/11 (9.1%)	0/25 (0.0%)	0/19 (0.0%)	0.1304

UTI: urinary-tract infection.

**Table 4:** Pulmonary pathogens identified pre- and postmortem. Numbers of isolates may not add up to the number of patients, as some patients had more than one organism, or no organism identified.

	Pre-Mortem Pulmonary Culture Results (*n* = 11)	Postmortem Sequencing Results (*n* = 36)
*P. aeruginosa*	4	2
*K. pneumoniae*	4	0
MSSA	3	2 *
MRSA	3	
*E. coli*	2	3
*A. baumanii*	2	1
*S. marcesens*	1	0
*K. aerogenes*	1	0
*K. oxytoca*	1	0
*P. mirabilis*	1	0
*C. albicans*	1	0
*Mycoplasma salivarium*	0	2
*Fusobacterium nucleatum*	0	2
*P. putida*	0	1
*S. pneumoniae*	0	1
*Legionella sp.*	0	1
*Prevotella melanogenica*	0	1

MRSA: methicillin-resistant Staphylococcus aureus. MSSA: methicillin-susceptible Staphylococcus aureus. * Includes all *S. aureus* as 16S PCR is unable to distinguish between MSSA and MRSA.

## Data Availability

The data presented in this study are available on request from the corresponding author. Individual patient data are not publicly available due to patient privacy.

## Supplementary Materials

The following supporting information can be downloaded at: https://www.mdpi.com/article/10.3390/pathogens12070932/s1. Figure S1: Chart-review form used to collect demographic and treatment data; Figure S2: Flow diagram illustrating sample-selection criteria and results of 16S bacterial PCR and sequencing on lung tissue; Table S1: Characteristics of autopsy cases separated by site of autopsy; Table S2: NGS sequencing reports by patient; Table S3: Comparison of cases with pulmonary co-infection with and without fungal co-infection.
