# Supplementary material for: Pulmonary Co-Infections Detected Premortem Underestimate Postmortem Findings in a COVID-19 Autopsy Case Series
Source: Pathogens. 2023 Jul 12;12(7):932. doi: 10.3390/pathogens12070932 (PMC10383307; doi:10.3390/pathogens12070932)
Supplement: Supplementary file 1 [file pathogens-12-00932-s001.zip › pathogens-2433984-supplementary.pdf]

Supplementary Material

Patient #	Patient Name	Hospital	Sex	DOB	Age at Death	Race	Ethnicity	BMI	Symptom Onset

Hospital Admission	Did this patient undergo multiple hospitalizations?	Are outside hospital records available on this patient, either within the hospital system or CRIS via EMR?	Date of Death	Time of Death	Date of Autopsy	Time of Autopsy	Medical History

Surgical History	Enter all documented Home Medications	List any vasopressive medications received	List any antibiotics received	List any systemic steroids received	List any prophylactic anticoagulation	List any system anticoagulation received	List any paralytics received

List any inhaled vasodilators received	List COVID-specific treatments received	Was this patient placed on ECMO?	List any form of Renal Replacement Therapy patient received	Was this Patient Intubated?	Did this patient undergo tracheostomy?	If patient was not intubated, list all forms of respiratory care patient received	Did patient have chest tube(s) placed?

Supplementary Figure S1: Chart review form used to collect demographic and treatment data.

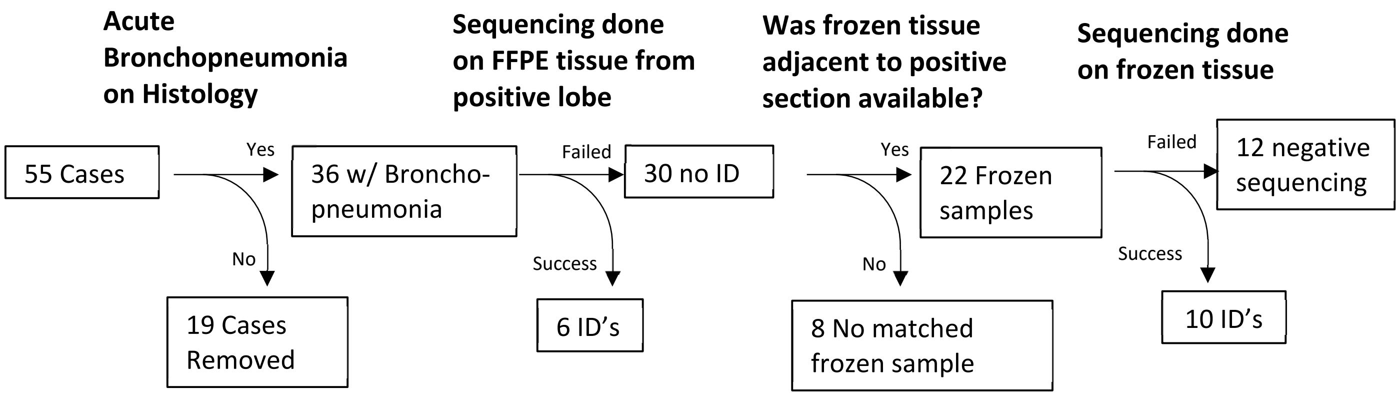

Supplementary Figure S2: Flow diagram illustrating sample selection criteria and results of 16S bacterial PCR and sequencing on lung tissue.

Supplementary Table S1: Characteristics of autopsy cases separated by site of autopsy. P values are univariate analyses between sites.

	**NIH (n=44)**	**UW (n=11)**	**p value**
Median age in years (IQR)	62.5 (47.25,71)	63 (53,72)	0.8659
Female, %	31.80%	36.40%	0.9999
Race, %			0.0968
White	43.20%	54.50%	
Black or African American	40.90%	9.10%	
Hispanic Ethnicity, %	15.90%	36.40%	0.9999
Median BMI (IQR)	30.95 (25,36.9025)	32.75 (27.3,38.15)	0.6199
Comorbidities			
COVID-related (IQR)	2 (1,3)	3 (2,4)	0.0867
Days from onset to death (IQR)	21 (12,37.5)	18 (16,22.5)	0.4442
Hospital duration, days (IQR)	13 (6,30.25)	13 (6.5,20.5)	0.7281
ICU duration, days (IQR)	11 (4,26.5)	13 (6,18)	0.9865
Intubated, %	79.50%	100.00%	0.1787
Intubation duration, days (IQR)	11 (3,28)	12 (2,18)	0.5642
Post-Mortem Interval, hours (IQR)	22.2 (18.2,33.9)	72 (36,84)	<0.0001
Presence of Bronchopneumonia, (%)	65.10%	63.60%	0.9999

Supplementary Table S2: Premortem and postmortem pulmonary co-infection results. Cases where premortem culture results align with postmortem sequencing are in red. To define semi-quantitative abundance in NGS sequencing three synthetic control DNA templates were spiked into reactions at defined copy numbers (2000, 200, and 20), with "major abundance" indicating organism-specific reads exceeding the most abundant synthetic template; "minor abundance" indicating those below the least abundant synthetic template, and "moderate abundance" falling between those levels.

Patient ID	Premortem pulmonary co-infection diagnosed	Premortem respiratory pathogens cultured	Antibiotics received	Postmortem broncopeum noia present	B&H stain positive	GMS stain positive	Sequencing Results
P1			Linezolid, pipperacillin-tazobactam, ceftriaxone, vancomycin, clindamycin	Yes	Yes	Yes	[Major abundance]: Prevotella melaninogenica<br><br>[Moderate abundance]: Mycoplasma salivarium, Fusobacterium nucleatum<br><br>[Minor abundance]: Prevotella oulorum, Limosilactobacillus (Lactobacillus) fermentum
P2			vancomycin, cefepime				[Major abundance]: Pseudomonas aeruginosa<br><br>[Moderate abundance]: Enterococcus faecalis
P3			vancomycin				[Major abundance]: Pseudomonas aeruginosa<br><br>[Moderate abundance]: Bacteria of the Family Enterobacteriaceae
P4					Yes		[Moderate abundance]: Corynebacterium striatum, Staphylococcus aureus, Streptococcus salivarius group, Rothia mucilaginosa<br><br>[Minor abundance]: Streptococcus parasanguinis, Corynebacterium propinquum, Veillonella parvula, Veillonella species, Granulicatella adiacens, Streptococcus mitis group (not Streptococcus pneumoniae), Streptococcus gordonii, Staphylococcus epidermidis, Streptococcus mutans, Actinomyces gerencseriae, Corynebacterium accolens
P5	Yes	K. pneumoniae, S. marcescens	Ceftriaxone, doxycycline, linezolid, piperacillin-tazobactam, vancomycin, amikacin, meropenem, polymyxin B, micafungin	Yes		Yes	[Major abundance]: Staphylococcus aureus<br><br>[Minor abundance]: Pseudomonas putida group
P6			Piperacillin-tazobactam, vancomycin	Yes			Minor abundance]: Mycoplasma salivarium, Fusobacterium nucleatum
P7			amikacin, cefepime, meropenem, micafungin, vancomycin	Yes	Yes		[Major abundance]: Lactobacillus gasseri<br><br>[Moderate abundance]: Prevotella oris, Prevotella scopos<br><br>[Minor abundance]: Prevotella jejuni, Streptococcus anginosus

P8	Yes	MSSA, A. baumanii	amoxicillin, ampicillin-sulbactam, cefazolin, cefepime, ceftriazone, linezolid, meropenem, minocycline, polymixin B	Yes	Yes		[Minor abundance]: Streptococcus pneumoniae, Neisseria subflava, Porphyromonas pasteri, Fusobacterium periodonticum or Fusobacterium pseudoperiodonticum, Porphyromonas species, Gemella sanguinis, Phocaeicola (Bacteroides) dorei, Haemophilus parainfluenzae, Streptococcus salivarius group
P9	Yes	K. aerogenes, P. aeruginosa	Azithromycin, amikacin, ampicillin, cefazolin, cefapime, fluconazole	Yes	Yes		[Moderate abundance]: Prevotella oris<br><br>[Minor abundance]: Prevotella jejuni, Veillonella species, Veillonella atypica, Prevotella melaninogenica, Staphylococcus aureus, Lactobacillus gasseri
P10			none	Yes	Yes		[Moderate abundance]: Bacteria of the Order Bacteroidales, Bacteria of the Phylum Bacteroidetes<br><br>[Minor abundance]: Prevotella melaninogenica, Prevotella nanceiensis
P11	Yes	P. aeruginosa, E. coli	amikacin, cefepime, daptomycin, meropenem, metronidazole, vancomycin, linezolid, azithromycin, ceftriaxone	Yes	Yes		
P12	Yes	A. baumanii, MRSA, K. pneumoniae	ampicillin-sulbactam, ceftriaxone, meropenem, minocycline, polymyxin B, linezolid, piperacillin-tazobactam, cefepime, levofloxacin, micafungin, vancomycin	Yes			
P13	Yes	K. pneumoniae, P. aeruginosa, K. oxytoca	ceftolozane-tazobactam, linezolid, cefepime, amikacin, ampicillin, meropenem, ceftriaxone	Yes			
P14			piperacillin/tazobactam, vancomycin, doxycycline	Yes	Yes		
P15			levofloxacin, imipenem/cilastin, ceftriaxone	Yes	Yes	Yes	
P16			vancomycin, piperacillin-tazobactam		Yes		
P17	Yes	MRSA, MSSA	vancomycin, ceftaroline, daptomycin, piperacillin-tazobactam, micafungin	Yes	Yes		

P18			aztreonam, vancomycin	Yes	Yes
P19			levofloxacin, vancomycin	Yes	
P20	Yes	E. coli, P. aeruginosa	cefepime, ertapenem, meropenem, amikacin, ceftolozane-tazobactam, linezolid, fluconazole, vancomycin	Yes	
P21			vancomycin, piperacillin-tazobactam		
P22			none		
P23			ceftriaxone, azithromycin		Yes
P24			ceftazidime, aztreonam, clindamycin, imipenem-cilastin, vancomycin	Yes	Yes
P25			azithromycin, ceftriaxone, ampicillin-sulbactam, doxycycline	Yes	Yes
P26			ceftriaxone, azithromycin, cefepime, piperacillin-tazobactam, vancomycin, linezolid		
P27			vancomycin, piperacillin-tazobactam	Yes	Yes
P28			none		
P29			piperacillin-tazobactam, sulfamethoxazole-trimethoprim, amikacin, amphotericin B, ceftriaxone, fluconazole, micafungin, tobramycin, valgancyclovir, vancomycin, voriconazole		
P30			micafungin, piperacillin-tazobactam, vancomycin	Yes	Yes
P31			azithromycin, ceftriaxone, linezolid, piperacillin-tazobactam	Yes	Yes
P32			none		

P33			none	Yes	Yes	
P34			vancomycin, piperacillin-tazobactam, clindamycin			
P35	Yes	P. aeruginosa	vancomycin, piperaciilin-tazobactam, ceftriaxone			
P36			piperacillin-tazobactam	Yes	Yes	
P37			piperacillin-tazobactam, vancomycin, meropenem, ampicillin-sulbactam	Yes	Yes	
P38			azithromycin, cefazolin, meropenem	Yes	Yes	
P39			amikacin, cefepime, linezolid, micafungin, piperacillin-tazobactam, voriconazole	Yes	Yes	Yes
P40	Yes	K. pneumoniae	piperacillin-tazobactam, ampicillin-sulbactam, vancomycin,	Yes		
P41	Yes	MSSA	ampicillin sulbactam, ceftriaxone, doxycycline,			
P42	Yes	P. aeruginosa	vancomycin, cefepime, metronidazole, fluconazole, trimethoprim-sulfamethoxazole , ceftriaxone, meropenem, piperacillin-tazobactam, daptomycin, micafungin, amikacin, ceftazidine-avybactam			
P43			ceftriaxone, azithromycin	Yes	Yes	
P44	Yes	MSSA	ceftriaxone, azithromycin, ampicillin, cefazolin, cefepime, fluconazole, gentamicin, linezolid, meropenem, micafungin, piperacillin-tazobactam, vancomycin	Yes	Yes	Yes

AU-10			none		
AU-13			cefepime, piperacillin-tazobactam, ampicillin	Yes	Yes
AU-17			azithromycin, vancomycin, cefepime, metronidazole	Yes	Yes
AU-18			vancomycin, ceftazidime		
AU-19			ceftriaxone, doxycycline	Yes	
AU-20			vancomycin	Yes	Yes
AU-21	Yes	MRSA	vancomycin, meropenem, doxycycline	Yes	
AU-22			vancomycin, cefepime	Yes	
AU-29			vancomycin, ceftriaxone	Yes	Yes
AU-30	Yes	E. cloacae, K. pneumoniae, MSSA	vancomycin, meropenem, doxycycline, voriconazole		
AU-31			ceftriaxone, vancomycin		

Supplementary Table 3: Comparison of cases with pulmonary co-infection with and without fungal co-infection. P values are univariate analyses between groups.

	Fungal Pulmonary Co-infection (n=5)	Bacterial Only Co-infection (n=31)	p-value
**Median age in years (IQR)**	41 (27,61)	63 (58,70.5)	0.0480
**Female (%)**	0/5 (0.0%)	12/31 (38.7%)	0.1464
**Race (%)**			0.5114
**White (%)**	3/5 (60.0%)	14/31 (45.2%)	
**Black or African American (%)**	1/5 (20.0%)	11/31 (35.5%)	
**Hispanic Ethnicity (%)**	2/5 (40.0%)	6/31 (19.4%)	0.3048
**Median BMI (IQR)**	39.2 (32.0,51.0)	31.7 (26.3,38.5)	0.2616
**Major Comorbidities (IQR)**	1 (1,1)	2 (2,4)	0.0273
**Immunosuppression/Cancer (%)**	0/5 (0.0%)	5/31 (16.1%)	0.9999
**Pulmonary (%)**	0/5 (0.0%)	12/31 (38.7%)	0.1464
**Cardiac (%)**	1/5 (20.0%)	21/31 (67.7%)	0.0637
**Liver (%)**	0/5 (0.0%)	2/31 (6.5%)	0.9999
**Neuro (%)**	0/5 (0.0%)	3/31 (9.7%)	0.9999
**Diabetes Mellitus (%)**	1/5 (20.0%)	15/31 (48.4%)	0.3549
**CKD (%)**	0/5 (0.0%)	5/31 (16.1%)	0.9999
**Obesity (%)**	4/5 (80.0%)	18/31 (58.1%)	0.6283
**Days from onset to death (IQR)**	31 (27,42)	18 (10.5,26.5)	0.0453
**Hospital duration, days (IQR)**	27 (22,33)	10 (5.5,16.5)	0.0016
**ICU duration, days (IQR)**	22 (22,29)	7 (4,14.5)	0.0207
**Intubation duration, days (IQR)**	22 (21.0,29.0)	6 (1.0,13.5)	0.1076
**Post-Mortem Interval, hours (IQR)**	19.8 (19.2,33.7)	24.6 (19.5,36.5)	0.5296
**ICU Admission, (%)**	5/5 (100.0%)	29/31 (93.5%)	0.9999
**Intubated, (%)**	5/5 (100.0%)	27/31 (87.1%)	0.9999
**Pressor Use (%)**	5/5 (100.0%)	23/31 (74.2%)	0.5655

**RRT (%)**	3/5 (60.0%)	12/31 (38.7%)	0.6296
**ECMO (%)**	2/5 (40.0%)	5/31 (16.1%)	0.2440
**Abnormal Chest Imaging (%)**	5/5 (100%)	30/31 (96.8%)	0.9999
**Antibiotic Use, (%)**	5/5 (100.0%)	29/31 (93.5%)	0.9999
**HAP Coverage, (%)**	4/5 (80.0%)	20/31 (64.5%)	0.6457
**COVID-Specific Therapies**			
**Steroid Use, (%)**	5/5 (100.0%)	27/31 (87.1%)	0.9999
**Remdesivir, (%)**	4/5 (80.0%)	14/31 (45.2%)	0.3377
**Tocilizumab, (%)**	2/5 (40.0%)	2/31 (6.5%)	0.1548
**Convalescent Plasma, (%)**	2/5 (40.0%)	6/31 (19.4%)	0.3048
**Days from onset to death (IQR)**	31 (27,42)	18 (10.5,26.5)	0.0453
**Hospital duration, days (IQR)**	27 (22,33)	10 (5.5,16.5)	0.0016
**ICU duration, days (IQR)**	22 (22,29)	7 (4,14.5)	0.0207
**Intubation duration, days (IQR)**	22 (21.0,29.0)	6 (1.0,13.5)	0.1076
**Post-Mortem Interval, hours (IQR)**	19.8 (19.2,33.7)	24.6 (19.5,36.5)	0.5296
**ICU Admission, (%)**	5/5 (100.0%)	29/31 (93.5%)	0.9999
**Intubated, (%)**	5/5 (100.0%)	27/31 (87.1%)	0.9999
**Any Pre-mortem Infection**	4/5 (80.0%)	12/31 (38.7%)	0.1493
**Pulmonary**	2/5 (40.0%)	9/31 (29.0%)	0.6309
**Extra Pulmonary**	4/5 (80.0%)	11/31 (35.5%)	0.138
**Bacteremia**	3/5 (60.0%)	8/31 (25.8%)	0.1544
**UTI**	0/5 (0.0%)	2/31 (6.5%)	0.9999
**Fungemia**	2/5 (40.0%)	2/31 (6.5%)	0.0843

**Skin/Soft Tissue Infection**	1/5 (20.0%)	0/31 (0.0%)	0.1389
***C. difficile* colitis**	0/5 (0.0%)	1/31 (3.2%)	0.9999
